# Experimental study of hypoxia-induced changes in gene expression in an Asian pika, *Ochotona dauurica*

**DOI:** 10.1371/journal.pone.0240435

**Published:** 2020-10-12

**Authors:** Katherine A. Solari, Elizabeth A. Hadly

**Affiliations:** 1 Department of Biology, Stanford University, Stanford, California, United States of America; 2 Program for Conservation Genomics, Stanford University, Stanford, California, United States of America; 3 Woods Institute for the Environment, Stanford University, Stanford, California, United States of America; 4 Center for Innovation in Global Health, Stanford University, Stanford, California, United States of America; Smithsonian Conservation Biology Institute, UNITED STATES

## Abstract

Acclimation to environmental changes driven by alterations in gene expression will serve as an important response for some species facing rapid Anthropogenic climate change. Pikas, genus *Ochotona*, are particularly vulnerable to climate change and current trends suggest that only the highest, coldest elevations within their ranges may remain suitable habitat for these species. In this study we aimed to assess the role of changes in gene expression in potentially facilitating elevational movements in pikas by measuring gene expression in the only known captive pika population, *Ochotona dauurica*, in response to hypoxic conditions. Using a controlled experiment, we exposed four male pikas to oxygen concentrations characteristic of sea-level, 2,000 m, and 4,000 m for 5 days each. Using blood samples collected after each treatment, we used RNAseq to determine if candidate pathways were undergoing significant changes in gene expression at different levels of oxygen (~100%, ~77%, and ~61% of sea-level oxygen concentrations). Gene set enrichment analyses showed that gene sets associated with the oxidative phosphorylation pathway and electron transport chain were significantly enriched for up-regulated genes in the 4,000 m samples compared to samples from the same individuals at lower-elevation conditions. Up-regulation of these pathways is consistent with known mechanisms of oxygen compensation. Our results suggest that these pikas have the acclimation capacity to tolerate oxygen concentrations characteristic of any elevation within their species range and that gene expression can be changed in a matter of days to accommodate drastically different oxygen concentrations. Thus, rapid and radical elevational movements that may be required of some pika species to avoid warmer temperatures in the Anthropocene will likely not be limited by hypoxic stress.

## Introduction

In response to warmer climates the distributions of many species have been changing [1]. In general, flat-land species are shifting their ranges to higher latitudes and montane species are shifting their ranges to higher elevations [2] at rates that are too fast for evolutionary changes to keep pace [3, 4]. Contrary to genetic adaptions however, acclimation—physiological adjustments to changing environmental conditions [5] that is largely dependent on changes in gene expression—can act on much shorter time scales and can effectively buffer species from environmental change. There are many indications that acclimation, a type of phenotypic plasticity, will likely play a large role in how successfully species are able to respond to Anthropogenic climate change [6, 7]. However, acclimation capacity varies greatly between species [8].

One organism particularly vulnerable to climate change, with some populations already experiencing elevational range shifts at rates potentially ten times the global average, are pikas (Family Ochotonidae) [1, 9]. There are at least 28 species of extant pikas, all in the genus *Ochotona* [10–12]. Two species reside in North America and the rest live in Asia. All pika species are cold-specialists and are generally only found at high latitudes, high elevations, or in cold-microclimates. Pikas display a limited ability to acclimate to changes in temperature [13], largely relying on behavioral plasticity to avoid overheating [14, 15]. Correlated with increasing temperatures, the trailing edge of American pika populations in the Great Basin is moving up in elevation at a rate of 145 m per decade [9]. Asian pika species that occupy parts of the Himalayan mountain range and Tibetan Plateau (as many as 15 species) [16] are at even greater risk of range retractions because this part of the world is experiencing temperatures changes three times faster than the global average [17].

The mechanism behind this movement upward of the lower-elevational limit of their range is unknown; it could be a result of lower-elevation populations moving into the higher elevations within their species range or extirpation of lower-elevation populations. If this is a result of the movement of low-elevation populations upward, these populations may encounter physiological stress from the decrease in oxygen found at higher elevations. Given the significant stress that high-altitude hypoxia puts on an organisms’ physiology [18], it is unsurprising that high-altitude organisms spanning a range of taxa have all shown distinct genetic adaptations in response to this stress [19–26]. Specifically, high-elevation pika species are known to have genetic changes in both hemoglobin and cytochrome c oxidase genes that may help them tolerate these low-oxygen environments [27–29]. However, there is mounting evidence that differences in gene expression may also play an important role in hypoxia tolerance in high-elevation pikas, with differences in gene expression distinguishing individual pikas occupying higher elevations within a population in the wild [30]. This suggests that while pikas appear to have a very limited ability to acclimate to changes in temperature, they may be able to acclimate to changes in oxygen. However, it is still unclear if differences in gene expression between pikas at different locations represent set differences between individuals or rather regulatory changes in gene expression that can occur within any individual.

To directly test the capacity to acclimate to changes in oxygen concentration within an individual, we worked with the only known captive population of pikas in the world, *Ochotona dauurica*, held at the Minnesota Zoo. *Ochotona dauurica* is found through much of Mongolia and also extends into Russia and China [31]. *Ochotona dauurica* occupies elevations from 400–4,000 meters in the wild [32], and thus in this experimental study we exposed captive pikas to oxygen concentrations characteristic of the low (close to sea-level), middle (~2,000 m), and high point (~4,000 m) of their natural range and collected blood samples after each treatment. Blood samples were used to characterize gene expression, blood composition, and physiology at each time point. By examining gene expression from the same individuals before and after exposure to different oxygen concentrations for extended periods of time while controlling for all other variables, we were able to directly test what role changes in gene regulation may play in facilitating the movement of a pika into a more hypoxic environment.

## Materials and methods

### Experimental design

**Fig 1:**
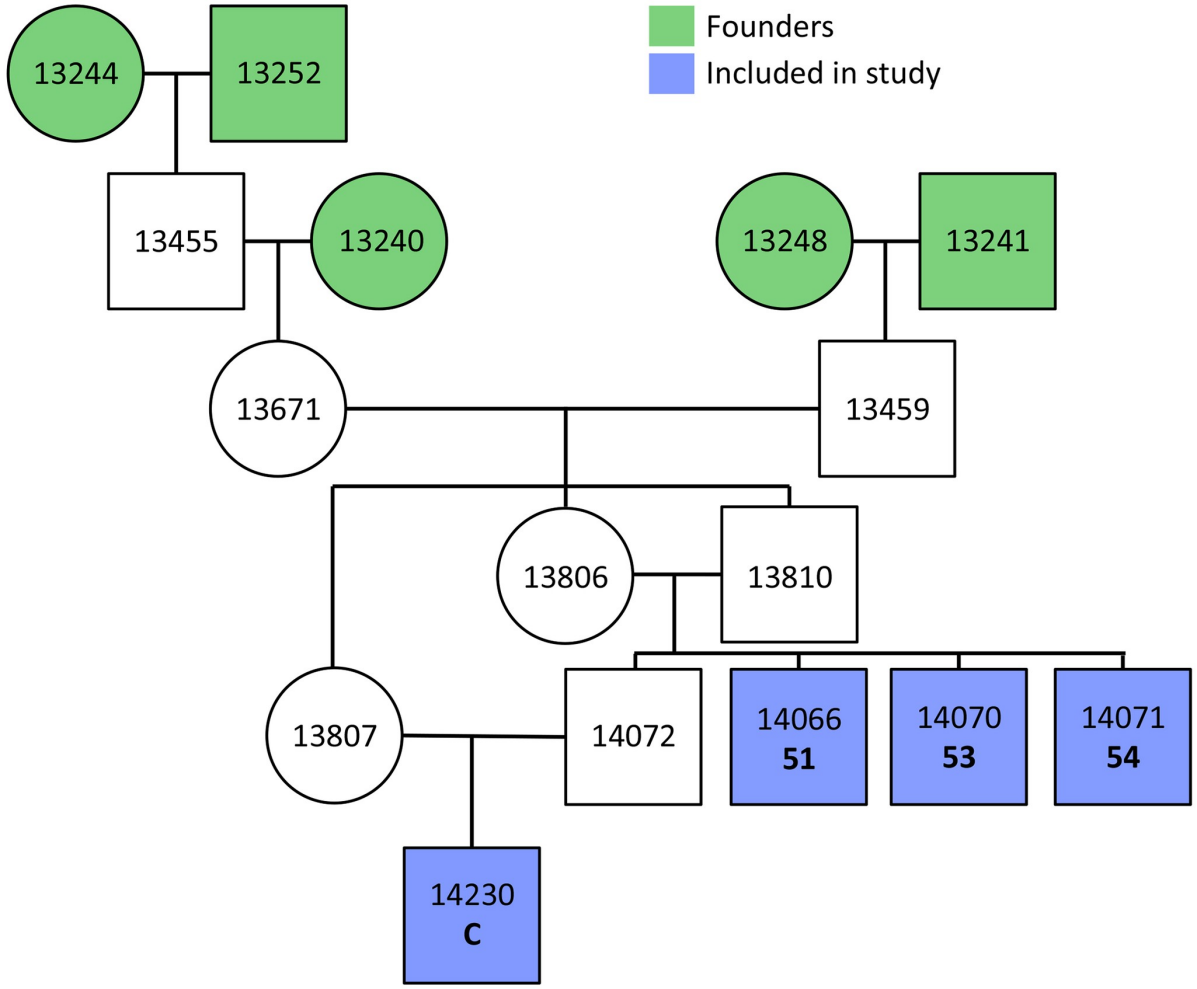
Family pedigree displaying relatedness of individuals used in this study. Wild individuals collected from Mongolia (founders) are shown in green. The four male pikas used in this study are shown in blue. Circles indicate females and squares indicate males. The inbreeding coefficient for individuals 51, 53, and 54 is 0.25. The inbreeding coefficient for individual C is 0.3125.

Fifteen *O*. *dauurica* individuals were collected in Mongolia near Ulan Bator and received by the Minnesota Zoo (elevation 310 meters) on December 18, 2011 where they have been maintained and bred since then at ambient oxygen concentration. Between August 5–27, 2015, three 4^th^ generation captive males (individual 51, 53, and 54, born between December 28, 2014 –January 3, 2015) and one 5^th^ generation captive male (individual C, born June 8, 2015) were sampled for this study (Fig 1).

All four male pikas used in this study were kept at the Minnesota Zoo in individual metal cages (20”x30”x10” high). Each cage had a wooden nest box, 6.25” x 8” x 6” high with a 2.25” diameter hole, that contained pinewood shavings (Dejno’s, Inc.) as bedding. All pikas were provided with ample water and fed 1–2 fresh dandelion leaves, 1–2 leaves of baby kale, a slice of carrot, 20 grams of chinchilla formula (Vitakraft Sunseed, Inc.) and orchard grass (Vitakraft Sunseed, Inc.) daily. Water and food intake of pikas, as well as their weight, was monitored daily to insure their health. A graph showing the weight of each pika throughout the experiment is given in S1 Fig. Temperature, humidity, and oxygen concentration in the tent were recorded twice daily. Pikas were kept on a 12-hour light, 12-hour dark cycle with lights on from 6 AM to 6 PM.

**Fig 2:**
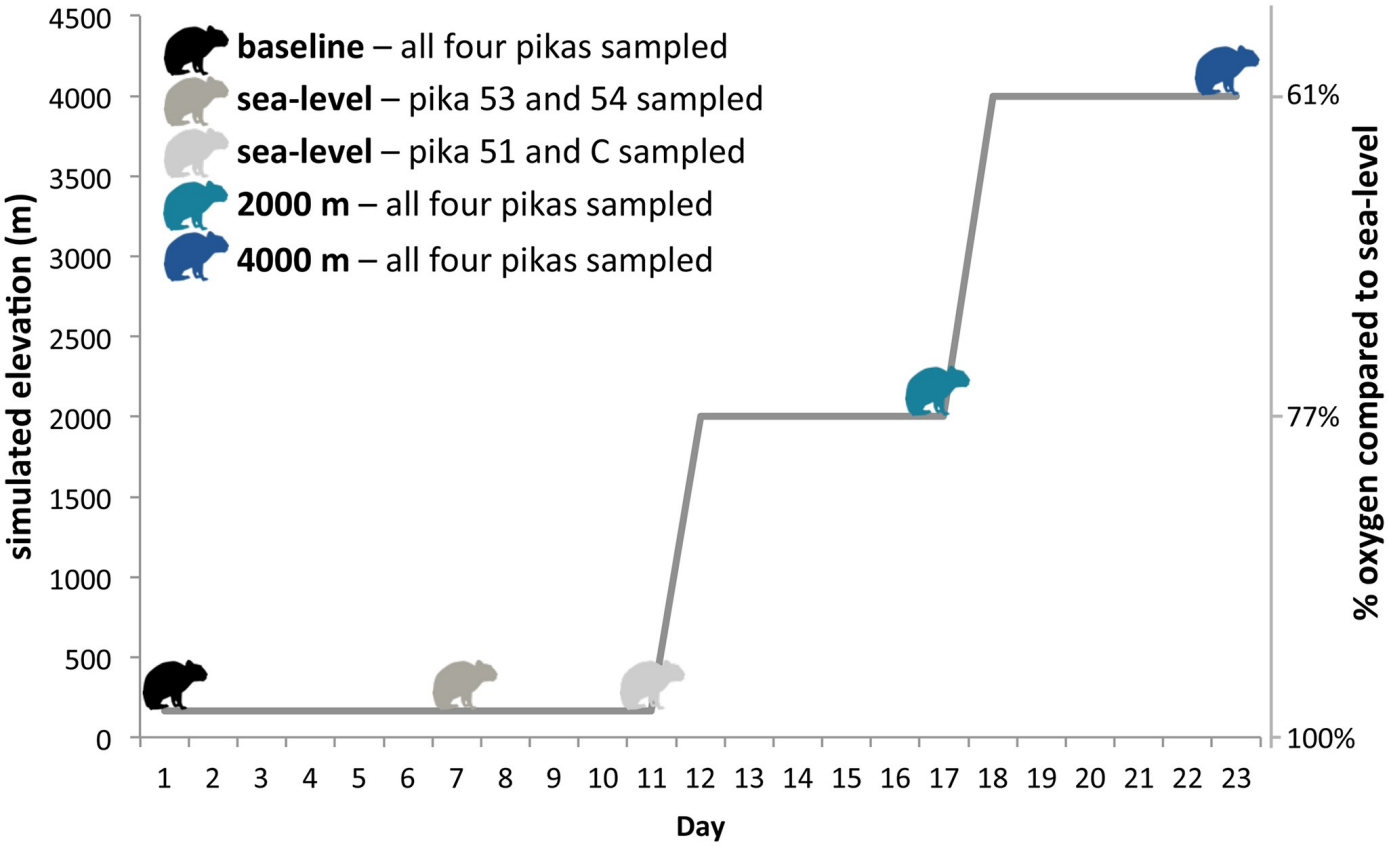
Sampling scheme indicating the simulated elevation and denoting when blood samples were collected.

During the experiment, all four pikas were sampled at four different time points. On day 1, the pikas were anesthetized using isoflourane (the inhalant anesthetic most commonly used when handling American pikas [33]) and the sex of each individual was confirmed and 300 μL of blood was collected from each individual using retro orbital abrasion (“baseline” samples). The four pika cages were then placed into a Hypoxico tent connected to an Everest Summit II hypoxic generator (Hypoxico Inc, New York, NY). For the first 11 days, the hypoxic generator was set to generate oxygen concentrations characteristic of sea-level. Two Handi oxygen monitors (Maxtec, Salt Lake City, UT) were used to confirm that the oxygen concentration in the tent was consistently between 21.0–20.8% (~100% oxygen compared to sea-level). Two pikas (individual 53 and 54) were successfully sampled again on day 7. The other two pikas (individual 51 and C), which we had difficulty drawing blood from on day 7, were successfully sampled on day 11. In both cases, pikas were anesthetized using isoflourane and 100–200 μL of blood was collected from each individual (“sea-level” samples). After sampling on day 11, the hypoxic generator was set to generate an oxygen concentration characteristic of ~2,000 m, and two Handi oxygen monitors were used to confirm that the concentration in the tent during this time was consistently between 16–16.3% (~77% oxygen compared to sea-level). On day 17, after over five days at this oxygen concentration, all four pikas were again anesthetized and blood samples were collected (“2,000 m” samples). On day 18, an adjustable high-altitude adapter was added to the hypoxic generator and it was set to generate an oxygen concentrations characteristic of ~4,000 m. During this time there was 12.7–13.0% oxygen in the tent (~61% oxygen compared to sea-level), confirmed multiple times a day using Handi oxygen monitors. On day 23, after over five days at this oxygen concentration, blood samples were collected (“4,000 m” samples). All blood samples after the initial sampling consisted of 100–200 μL of blood collected from the jugular vein or vena cava. In total, each of the four pikas were sampled four times, resulting in a total of 16 samples (Fig 2). Pikas were briefly removed from the tent for the blood sampling procedure. All samples were collected within 15 minutes of the pika being removed from the tent except for the sample from individual 54 at 2,000 m which was collected ~23 minutes after removal from the tent. After the final blood sample was taken on day 23, all of the pikas were removed from the tent and resumed being cared for by the Minnesota Zoo staff.

All blood samples were collected directly into Qiagen RNAprotect Animal Blood Tubes (Qiagen, Valencia, CA) following the manufacturer’s protocol in order to stabilize RNA. Samples were then stored in a -80°C freezer within two hours of collection. If possible, extra blood was collected and analyzed using a HemoCue Hb 201+ (HemoCue America, Brea, CA) and an i-STAT EC8+ (Abbott Point of Care, Inc., Princeton, NJ) in order to assess potential changes in hemoglobin concentration throughout the experiment, a response to hypoxia seen in numerous low-land species [34, 35]. When possible, blood smears were also made and analyzed by hand at the Stanford Medicine Veterinary Service Center Animal Diagnostic Laboratory. The percentage of lymphocytes, heterophils, monocytes, and eosinophils were measured from each blood smear, as well as an estimate of white blood cell concentration. Ratios of these different cell types can offer insights into the individual’s general health and stress level [36]. Significant differences in blood chemistry or composition between the time points were evaluated using t-tests in R.

This study and all procedures described were approved by the Stanford University Administrative Panel on Laboratory Animal Care (APLAC protocol 27547) and were conducted by licensed veterinary doctors or animal health specialists under the supervision of a veterinarian. Following all blood collections, the recovery of each pika was monitored by a veterinarian or animal health specialist.

Blood was targeted as a transcriptomically meaningful tissue that could be repeatedly collected with minimal harm to the animals. Blood has a relatively quick turnover rate and interacts with tissue throughout the body, offering real-time information of whole body status in response to physiological and environmental pressures [37–39]. Up to 61–80% of protein-coding genes have been found to be expressed in the blood transcriptome [37, 40, 41]. Indeed, previous studies have used the blood transcriptome to asses responses to high-altitude hypoxic stress in humans [42], the Tibetan Plateau saker falcon (*Falco cherrug*) [43], and a species of Himalayan pika (*Ochotona roylei*) [30]. These studies have generated meaningful results and confirm that the blood transcriptome is a promising indicator of hypoxic stress.

A previous elevational transplant experiment assessing changes in gene expression in the rufous-collared sparrow in the Andes found significant differences in gene expression using a 7-day acclimation period [44]. In humans, studies have found significant gene expression changes using blood samples taken two days after subjects were moved to high elevation from low elevation [42] as well as in blood samples taken only an hour after subjects were moved to low elevation from high elevation [45]. Given the results of these previous studies, we believed that allowing for a 5 day acclimation to each elevation prior to sampling was an adequate amount of time to observe changes in gene expression.

### RNA preparation and sequencing

Total RNA, excluding miRNA (less than 200 nucleotides in length), was extracted and purified from the RNAprotect-stabilized blood samples using the Qiagen RNeasy Protect Animal Blood Kit following the manufacture’s protocol to create a final 30 μL elution. RNA concentration of each sample was tested using a nanodrop and RNA integrity was checked on a bioanalyzer. Samples were then submitted to The California Institute for Quantitative Biosciences Functional Genomics Laboratory at the University of California, Berkeley for library preparations and sequencing. RNAseq libraries were made using PrepX RNA-seq sample and library preparation kit with Ribo depletion and sequenced on two lanes of Illumina 2500HiSeq 100-bp paired end and two lanes of Illumina 4000HiSeq 100-bp paired end. All samples were run in all lanes to avoid batch effects.

### RNA-Seq data processing and mapping to reference

Demultiplexed Illumina reads were corrected for random Illumina sequencing errors using Rcorrector with a *k*-mer size of 31 [46]. *Ochotona* spp. and *Microtus oeconomus* hemoglobin mRNA sequences were downloaded from Genbank (Accession numbers: XM_004589962.1, XM_004589963.1, XM_004596585.1, XM_004596781.1, XM_004589960.2, XM_004589961.2, XM_004596586.2, KC886314.1, KC886313.1, KC886312.1, KC886310.1, JX827174.1, JX827173.1, JQ968413.1, JQ968412.1, DQ839484.1, EF429202.1, JX827171.1, and JX827170.1) and made into a reference file using Bowtie2 [47]. All reads were then mapped to this hemoglobin mRNA reference using Bowtie2 and reads that mapped to the hemoglobin mRNA reference were removed from the sequence dataset. The blood transcriptome is dominated by hemoglobin mRNA, thus these sequences were removed to allow for accurate data quality assessment. Hemoglobin genes were not in any of the candidate gene sets tested in this study (described below) and thus remained excluded from downstream analyses. Reads were treated as single end to remove adapter sequences and low-quality reads were then removed using Trimmomatic [48]. Reads were then sorted, paired, and then sorted again using custom Python scripts. Read quality was checked before and after each filtering step using FastQC [49].

The *O*. *princeps* annotated genome and transcriptome were downloaded from NCBI (GCF_000292845.1_OchPri3.0 with 26,240 transcripts). *Ochotona princeps* is the closest relative to *O*. *dauurica* with an annotated genome and is approximately 15 million years diverged [50]. The complete mitochondrial genome from *O*. *curzoniae*, the most closely related pika species to *O*. *dauurica* with a mitochondrial genome, was also downloaded from Genbank (accession number EF535828.1). The 14 annotated genes from the *O*. *curzoniae* mitochondrial genome were extracted and added to the *O*. *princeps* reference transcriptome.

Filtered paired reads were mapped to the *O*. *princeps* nuclear transcriptome and *O*. *curzoniae* mitochondrial transcriptome using BWA-MEM [51]. Samtools v.0.1.19 [52] was used to sort mapped reads and remove duplicate reads. Raw counts were generated using a custom Python script. The hemoglobin alpha 1 transcript (XM_004596586.2) and the hemoglobin subunit beta transcript (XM_004589960.2) were removed to eliminate any additional reads mapping to hemoglobin mRNA. Raw read counts were normalized using the scaling factor normalization method included in the package DESeq V.1.22.1 [53] in R [54].

### Gene set enrichment analyses

Gene set enrichment analysis (GSEA) [55, 56] (http://www.broad.mit.edu/gsea/) is a computational method that can be used to determine if a predefined set of genes shows statistically significant differences in gene expression between a pair of sample sets. GSEA takes genome wide expression data for the two sets of samples that are being compared and ranks each transcript based on how correlated its expression profile is with its sample set identity. GSEA then tests each gene set to see if the transcripts in a set are randomly distributed across the ranked list of genes, or if they are located predominantly at the top or bottom of the ranked gene list, indicating that a gene set is enriched with up-regulated or down-regulated genes, respectively. The enrichment score (ES) of the gene set reflects the extent to which the gene set is over represented at the top or bottom of the ranked gene list. The p-value for the ES is calculated by either permuting the sample identity or by permuting gene sets to generate a null distribution. Permuting sample identity is favorable, as it preserves gene relationships; however, permuting by gene set is recommended by GSEA in cases were a sample set contains less than seven samples, as in the current study. ES are normalized based on the size of the gene set to generate a normalized enrichment score (NES). A false discovery rate (FDR) for each NES is calculated by comparing the observed and null distribution for each NES.

In this study, a gene set consists of the genes making up a given gene ontology (GO) category or Kyoto Encyclopedia of Gene and Genomes (KEGG) pathway. We drew candidate GO categories and KEGG pathways from previous studies that have explored changes in gene expression related to hypoxia in two other small mammal species, *O*. *roylei* (a Himalayan pika species) and *Peromyscus maniculatus* (deer mouse), the most closely related species for which any gene expression work related to hypoxia has been conducted. We used six gene sets that were up-regulated in high-elevation individuals of *O*. *roylei* when compared to lower-elevation individuals using blood samples [30]. We drew 11 gene sets from the four studies assessing changes in gene expression due to hypoxia in high and low-elevation deer mice using skeletal muscle and brown adipose tissue samples [57–60]. We also tested five gene sets that were likely to be related to hypoxia response based on their category or pathway description (Table 1).

**Table 1:** Gene sets used in GSEA.

GO category/KEGG pathway	Citation	# of transcripts
Oxidative phosphorylation (KEGG)	Pika and deer mouse [30, 57, 58]	168
Mitochondrial inner membrane (GO:0005743)	Pika [30]	595
Mitochondrial respiratory chain complex I assembly (GO:0032981)	Pika [30]	79
Mitochondrial electron transport, NADH to ubiquinone (GO:0006120)	Pika [30]	71
Mitochondrial respiratory chain complex I (GO:0005747)	Pika [30]	66
NADH dehydrogenase (ubiquinone) activity (GO:0008137)	Pika [30]	57
Lipid catabolic process (GO:0016042)	Deer mouse [57, 58]	347
Fatty acid β-oxidation pathway	Deer mouse [57, 58]	15
Regulation of skeletal muscle cell differentiation (GO:2001014)	Deer mouse [58]	30
Regulation of erythrocyte differentiation (GO:0045646)	Deer mouse [58]	56
Cellular response to reactive oxygen species (GO:0034614)	Deer mouse [58]	216
Water transport (GO:0006833)	Deer mouse [58]	22
Angiogenesis (GO:0001525)	Deer mouse [58–60]	588
Notch signaling pathway (GO:0007219)	Deer mouse [59]	239
Regulation of ERK1 and ERK2 cascade (GO:0070372)	Deer mouse [59]	342
Muscle structure development (GO:0061061)	Deer mouse [60]	980
Negative regulation of vascular permeability (GO:0043116)	Deer mouse [60]	15
Response to hypoxia (GO:0001666)	GO category definition	448
Cellular response to hypoxia (GO:0071456)	GO category definition	215
Response to oxidative stress (GO:0006979)	GO category definition	600
Cellular response to oxidative stress (GO:0034599)	GO category definition	380
HIF-1 signaling pathway (KEGG)	Pathway description	176

The 11 gene sets that we decide to include from the four studies assessing changes in gene expression due to hypoxia in high and low-elevation deer mice [57–60] were selected from dozens of gene sets that were identified across all four of these deer mouse studies. This long list of GO categories from the deer mouse studies where narrowed down to the 11 gene sets ultimately tested by 1) only considering GO categories that showed plasticity in expression within a population and that were associated with a measurement of hypoxia tolerance, 2) eliminating GO categories that had more than 1000 genes or less than 15 genes, and 3) using the REViGO on-line tool (http://revigo.irb.hr/) to collapse redundant GO categories [61].

Gene sets for each GO category were downloaded from AmiGO2 [62] using human annotation. Gene sets for KEGG pathways were downloaded from DBGET/LinkDB [63] using human annotation except for the gene set for the fatty acid β-oxidation pathway, which was taken from Cheviron et al. [57]. Genes that did not match a gene ID from the annotated transcriptome were investigated further and finalized gene set lists were curated by hand; however not all genes for every gene set were represented in the annotated pika transcriptome. Gene set permutations tend to result in more false positives, to account for this we used a more stringent FDR q-value cut-off of 5% for calling gene sets as differentially regulated as recommended by GSEA [55, 56]. The other parameters used in our GSEA analyses were set to the default. GSEA was used to test for differences between baseline and sea-level vs. 2,000 m, baseline and sea-level vs. 4,000 m, and all samples vs. 4,000 m. Heat maps summarizing the relative expression and mean normalized counts of each transcript identified as driving significant enrichment of gene sets were made using the package gplot v.3.0.1 in R v.3.2.3.

## Results

### RNA-seq data processing and mapping to reference

In this study, RNA-stabilized blood samples were collected from four male pikas held at the Minnesota Zoo at four time points (Fig 2). All four males were 4^th^ or 5^th^ generation captives and related to each other as indicated in Fig 1.

Illumina sequencing resulted in 19.39–138.03 (average 93.36) million reads per sample. Between 33–81% of the reads for each sample mapped to the hemoglobin mRNA reference and were removed from the sequence dataset. After all filtering steps were complete, there were 8.3–63.2 (average 25.5) million paired reads per samples. Between 33.4–58.0% (average 43.2%) of these reads from each sample mapped successfully to the *O*. *princeps* nuclear transcriptome and *O*. *curzoniae* mitochondrial transcriptome using BWA-MEM [51] with 18,961 transcripts out of the 26,254 (72% of the transcripts) represented by more than an average of 5 reads per sample after normalization. Removal of reads mapping to the hemoglobin alpha 1 transcript (XM_004596586.2) and the hemoglobin subunit beta transcript (XM_004589960.2) comprised 0.18–1.6% and 1.3–9.6% of the reads for each sample, respectively, leaving 0.7–6 (average 2.7) million reads per sample that successfully mapped to the reference transcriptome.

### Gene set enrichment analyses

We used GSEA to specifically test for enrichment in GO categories and KEGG pathways likely to be involved in response to hypoxia based on previous studies of pikas and deer mice.

**Fig 3:**
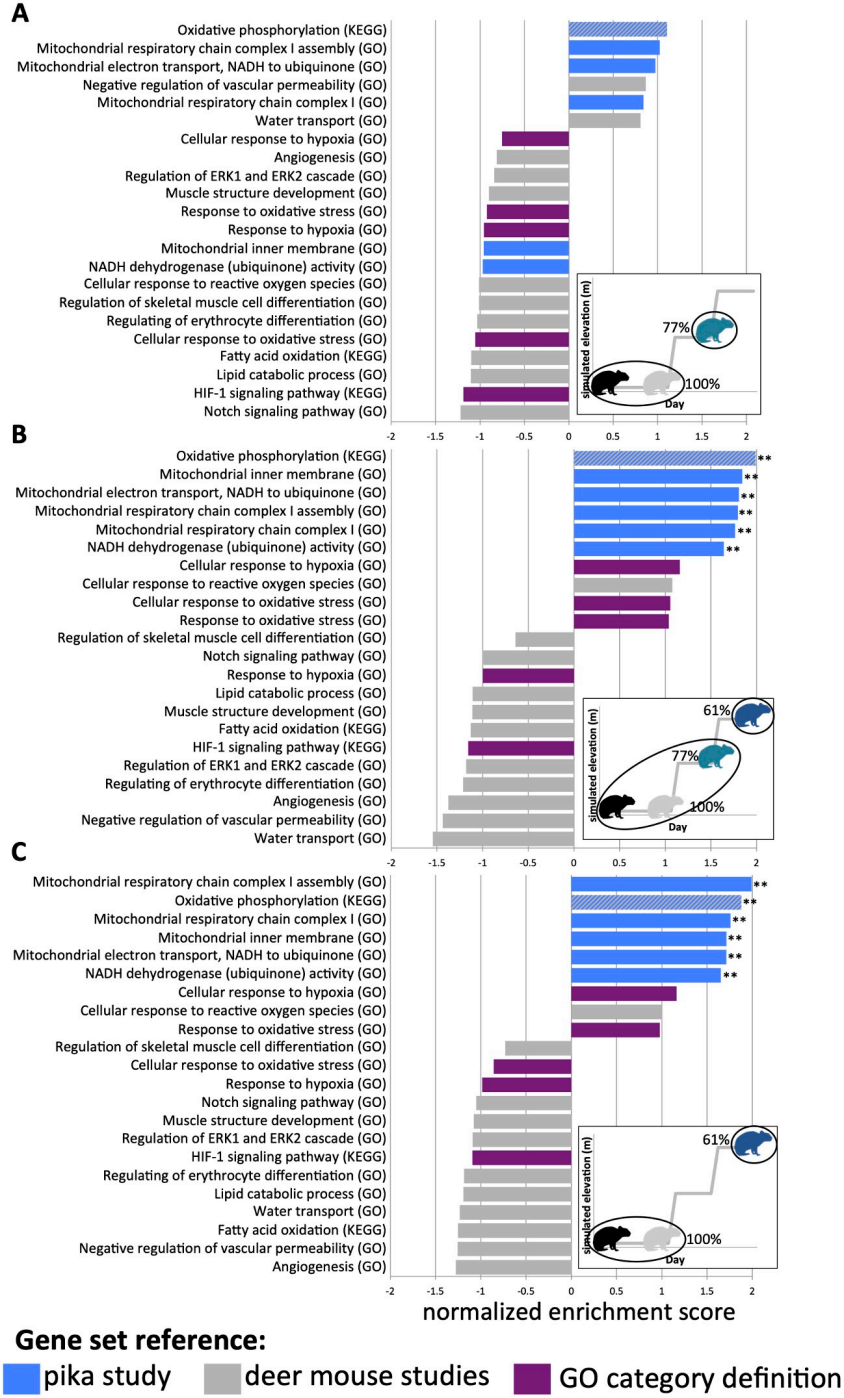
GSEA normalized enrichment score for each gene set. A diagram in the lower right of each panel indicates which sampling groups are being compared, with the percent oxygen compared to sea level of each sample group indicated. In all comparisons, the higher-elevation group is being compared to the lower-elevation group(s), so positive normalized enrichment scores indicate gene sets that are positively enriched in the higher-elevation samples compared to the lower-elevations samples. A) 2,000 m samples (77% oxygen compared to sea-level) compared to baseline and sea-level samples. B) 4,000 m samples (61% oxygen compared to sea-level) compared to all other samples. C) 4,000 m samples compared to baseline and sea-level samples. Gene sets drawn from the pika study are shown in blue, gene sets drawn from the deer mouse studies are shown in grey, and gene sets included due to their GO category definition are shown in purple. The oxidative phosphorylation pathway, which is drawn from both the pika and deer mouse studies is shown in blue and grey stripes. FDR q-values are indicated (**p<0.01, *p<0.05).

When we compared baseline and sea-level samples to 2,000 m samples, we found no gene sets to be enriched for up-regulated or down-regulated genes (Fig 3A, S1 Table). However, all six gene sets that were drawn from the *O*. *roylei* field study (gene sets up-regulated in the high-elevation *O*. *roylei* individuals compared to low-elevation individuals) were also significantly enriched in the 4,000 m samples when compared to all of the other samples (q-value for all six categories < 0.0083) (Fig 3B, Table 2, S2 Table) or just the baseline and sea-level samples (q-value for all six categories < 0.0046) (Fig 3C, Table 2, S3 Table). None of the gene sets drawn from the deer mouse studies or based on category descriptions showed significant enrichment in any of these comparisons (Fig 3B and 3C).

**Table 2:** Summary of all significant GSEA results.

Gene set	0 m vs. 2000 m	All vs. 4000 m	0 m vs. 4000 m	All vs. 4000 m (no 54)	0 m vs. 4000 m (no 54)
Oxidative phosphorylation (KEGG)		+**	+**	+*	
Mitochondrial inner membrane (GO)		+**	+**	+*	+*
Mitochondrial electron transport, NADH to ubiquinone (GO)		+**	+**		
Mitochondrial respiratory chain complex I assembly (GO)		+**	+**	+*	
Mitochondrial respiratory chain complex I (GO)		+**	+**		
NADH dehydrogenase (ubiquinone) activity (GO)		+**	+**		

Comparisons that showed significant positive enrichment scores in the higher-elevation group are indicated with a “+”. FDR q-values are indicated (**p<0.01, *p<0.05).

The subset of genes that account for the gene set’s enrichment signal are called the leading-edge genes. In the 4,000 m vs. all other samples comparison, there is substantial overlap in the leading edge genes among the 6 gene sets drawn from the pika study (S2 Fig).

The heat map of gene expression for leading-edge genes for the 4,000 m vs. all other samples comparison showed there to be a noticeably higher up-regulation of numerous genes in sample 54 at 4,000 m than the other 4,000 m samples (S3 Fig). However, even when removing this sample (S4 Fig) we found three of the gene sets to remain significantly enriched for up-regulated genes in the 4,000 m samples (not including sample 54) compared to all of the other samples (mitochondrial respiratory chain complex I assembly, q = 0.016; mitochondrial inner membrane, q = 0.022; and oxidative phosphorylation pathway, q = 0.047) (Table 2, S5 Fig, S4 Table). Additionally, when comparing the 4,000 m samples (not including 54) to the baseline and sea-level samples, the mitochondrial inner membrane gene set remained significant (q = 0.036) (Table 2, S5 Fig, S5 Table).

In our previous *O*. *roylei* field study [30], the up-regulation of the six gene sets used in this study were driven by the significant up-regulation of six nuclear genes (ATP5I, UQCRQ, NDUFA11, NDUFB7, NDUFS8, and NDUFS7) and two mitochondrial genes (CYTB and ND4). Five of these six nuclear genes also showed core enrichment in this study. In fact, the most differentially expressed gene in the oxidative phosphorylation (OXPHOS) pathway is the same in both studies—ATP5I. NDUFA11, the third most differentially expressed gene in the OXPHOS pathway in this study, was also fourth most differentially expressed in the pika field study. NDUFB7 and NDUFS7 were also in the top 13 most differentially expressed genes in the OXPHOS pathway in the present study.

### Blood measurements

Hemoglobin (Hb) concentrations were calculated from 10 samples using the HemoCue and 9 of these samples were also run on the i-stat device (Table 3). Some blood samples were not of large enough volume to allow for blood measurements. T-tests conducted in R showed no statistically significant differences in any of the i-stat or HemoCue results between sampling groups, likely partially due to limited statistical power because of the small sample size.

**Table 3:** Sampling information and blood measurement results.

					HemoCue results	I-stat results							Blood smear results				
Pika	Time point	Collection date (mm/dd/yy)	SRA accession numbers	weight (g)	Hb (g/dl)	Na (mmol/L)	K (mmol/L)	Cl (mmol/L)	BUN (mg/dL)	Glu (mg/dL)	Hct (%PCV)	Hb (via Hct) (g/dL)	% lymphocytes	% heterophils	% monocytes	% eosinophils	WBC estimate per μL
C	baseline	8/5/15	SAMN07155443	105	-	-	-	-	-	-	-	-	**-**	**-**	-	-	-
	sea-level	8/15/15	SAMN07155447	98	14.7	135	2.4	88	15	175	40	13.6	**78**	**22**	0	0	8571
	2000 m	8/21/15	SAMN07155451	104	16.1	137	2.8	91	15	147	46	15.6	**51**	**46**	3	0	1300
	4000 m	8/27/15	SAMN07155455	108	18	139	6	93	26	117	54	18.4	**54**	**42**	3	1	3583
51	baseline	8/5/15	SAMN07155444	146*	-	-	-	-	-	-	-	-	**-**	**-**	-	-	-
	sea-level	8/15/15	SAMN07155448	142	14.5	135	3.4	95	15	132	40	13.6	**82**	**15**	3	0	2500
	2000 m	8/21/15	SAMN07155452	134	14.7	156	6.9	90	23	103	43	14.6	**68**	**28**	4	0	2333
	4000 m	8/27/15	SAMN07155456	134	-	-	-	-	-	-	-	-	**-**	**-**	-	-	-
53	baseline	8/5/15	SAMN07155445	118*	-	-	-	-	-	-	-	-	**-**	**-**	-	-	-
	sea-level	8/11/15	SAMN07155449	132	-	-	-	-	-	-	-	-	**-**	**-**	-	-	-
	2000 m	8/21/15	SAMN07155453	122	13.3	137	3.5	97	17	155	35	11.9	**31**	**59**	8	2	3100
	4000 m	8/27/15	SAMN07155457	120	13.2	-	-	-	-	-	-	-	**60**	**38**	2	0	1136
54	baseline	8/5/15	SAMN07155446	116*	-	-	-	-	-	-	-	-	**-**	**-**	-	-	-
	sea-level	8/11/15	SAMN07155450	144	14.8	133	5.5	92	18	51	40	13.6	**83**	**14**	3	0	1929
	2000 m	8/21/15	SAMN07155454	138	13.9	143	5.8	95	16	224	38	12.9	**55**	**41**	1	3	1667
	4000 m	8/27/15	SAMN07155458	136	16.9	137	5.1	98	16	120	47	16	**48**	**51**	1	0	4121

Hb = Hemoglobin, Na = Sodium, K = Potassium, Cl = Chloride, BUN = Urea nitrogen, Glu = Glucose, Hct = Hematocrit, PCV = packed cell volume, WBC = white blood cell. Percent lymphocytes and percent heterophils, the only measures to be significantly different between sampling points, are shown in bold.

*weights measured 48 hours after sampling

**Fig 4:**
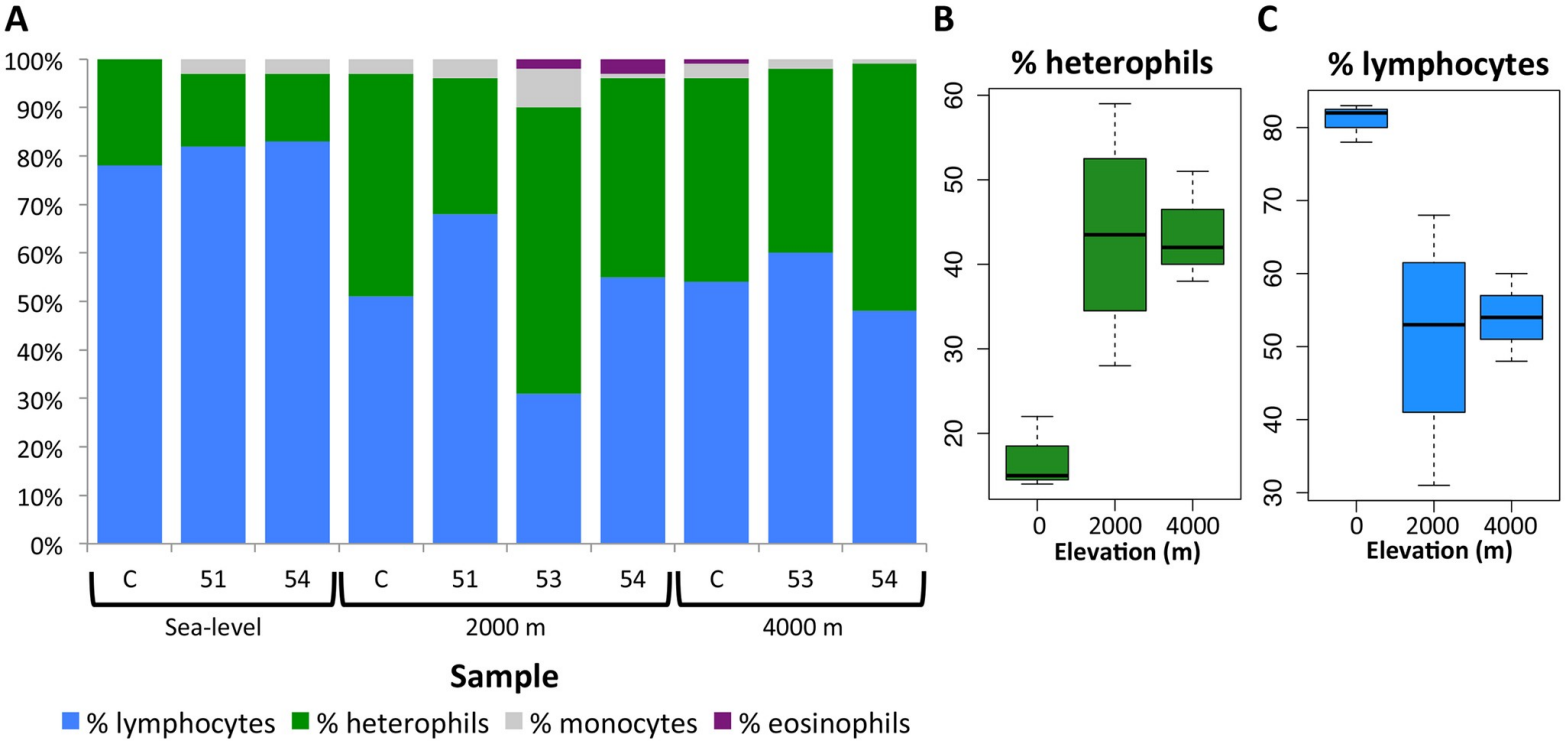
White blood cell composition calculated from blood smears. A) The composition of white blood cells for each of the 10 samples for which we have blood smears. B) Box-plot showing heterophil percentage grouped by elevation. C) Box-plot showing lymphocyte percentage grouped by elevation.

Blood smears were successfully collected for 10 of the samples: 3 from sea-level samples, 4 from 2,000 m samples, and 3 from 4,000 m samples. Blood smear results indicate that pikas have heterophils (a type of white blood cell), as are found in birds and rabbits, rather than functionally equivalent neutrophils, as are found in most all other mammals. Additionally, occasional Howell-Jolly bodies, small remnant clusters of DNA in red blood cells, were found in every blood smear. Occasional anisocytosis, red blood cells of unequal size, were observed in sample 54 at 4,000 m. The percentage of lymphocytes, heterophils, monocytes and eosinophils, as well as the white blood cell concentration estimate for each sample is given in Table 3. T-tests in R showed that the heterophil percentage and lymphocyte percentage in the sea-level samples were significantly different from the percentages in the 2,000 m and 4,000 m samples, p = 0.00039 and p = 0.0038, respectively (Fig 4).

## Discussion

Our results demonstrate that pikas are capable of significant changes in gene regulation, indicating substantial acclimation capacity, within an individual in response to hypoxic stress. Additionally, all the gene sets that were up-regulated in the highest elevation individuals in the previous field study of *O*. *roylei* [30] were significantly enriched for up-regulated genes in the 4,000 m samples in the current study of *O*. *dauurica*. This study also supports the findings of Solari et al. [30] that blood is a transcriptomically meaningful tissue that can be used as an alternative to lethal sampling which is used in similar transcriptomic studies [44, 57, 60].

The six gene sets that were drawn from the *O*. *roylei* field study [30], and that were also found to be significantly enriched for up-regulated genes in the 4,000 m samples in this study, are related to the OXPHOS pathway and electron transport chain. These gene sets are all related and thus have many overlapping genes (S2 Fig), so the up-regulation of each gene set is not independent. Thus, it is not surprising that we see all six gene sets up-regulated together in many of the comparisons. The OXPHOS pathway creates 95% of the cell’s energy [64] through the electron transport chain, a process that is pivotal for maintaining the health of the cell. This process is also highly dependent on oxygen availability (oxygen is used as a terminal electron acceptor) meaning that hypoxia can directly affect cellular viability through the OXPHOS pathway [65]. With oxygen availability positioned to put a direct selective force on this pathway, there have been numerous examples of selection in genes in this pathway in hypoxia-adapted species [21, 29, 66, 67]. Thus, up-regulation of genes in these gene sets is not only consistent with what we observed in the previous pika study, but is also consistent with a known avenue of hypoxia compensation observed across the mammalian tree. Additionally, these were the only gene sets to be significantly enriched for up-regulated genes in any comparisons.

The consistency of these results from *O*. *dauurica* with the previous study on *O*. *roylei*, two species that are approximately 15 million years diverged [50], suggests that the pathways used to compensate for oxidative stress in pikas have been conserved through the evolutionary history of the genus, and that such plasticity may be found across most pika species. Conversely, our results suggest that deer mice, and perhaps all rodents, which diverged from pikas more than 60 millions year ago [68], possess a different suite of transcriptomic responses to hypoxia.

Interestingly, in the current study of *O*. *dauurica*, which occupies elevations from 400–4,000 m [32], significant changes in gene expression were observed between 2,000–4,000 m. However, in the previous study of *O*. *roylei*, which occupies elevations from 2,400–5,000 m [69], we only observed significant changes in gene expression between 4,000–5,000 m. This may indicate that while these species use similar pathways to compensate for hypoxic stress, the up-regulation of these pathways may be induced by different levels of hypoxic stress in different species, perhaps depending on the maximum elevation that the species occupies, and perhaps due to tradeoffs the species encounters in the field that we could not measure. For example, *O*. *dauurica* and *O*. *roylei* differ in numerous ways–*O*. *dauurica* is a burrow-dwelling social pika species, while *O*. *roylei* is a rock-dwelling and relatively asocial species with lower fecundity [70].

Solari et al. [30] concluded that the pikas they sampled along one mountain in Spiti Valley, Himachal Pradesh, India were likely one population, as no evidence of significant population genetic structure was found. However, they were unable to conclude if observed differences in gene expression between high and lower-elevation pikas were due to within-individual changes in expression, and/or genetic or epigenetic differences. The results of this experimental study indicate within-individual changes in expression in *O*. *dauurica* and show that it is possible, if not likely, that the observed differences in *O*. *roylei* expression observed in the field are due to within-individual plasticity as well. The conservation of the pathways up-regulated in both species indicates a conserved response to hypoxia between these species, and thus suggests to us that the mechanism of up-regulation is likely conserved as well, although a similar experimental gene expression study of *O*. *roylei* would be needed to confirm this hypothesis.

We also found substantial overlap between the OXPHOS pathway genes most differentially expressed in our previous *O*. *roylei* field study [30] and those most differentially expressed in the current study. This overlap may indicate that not only is the same pathway being used to compensate for hypoxic stress in both of these pika species, but they both seem to be altering that pathway in a similar way. ATP5I, the gene in the OXPHOS pathway most up-regulated at high elevations in both pika studies, encodes subunit e of ATP synthase (complex V of the OXPHOS pathway). ATP synthase occurs in rows of dimers and this is what creates the curved shape of mitochondrial cristae [71]. In human cells, subunit e is pivotal in joining two ATP synthase complexes into a dimer and a down-regulation of ATP5I results in deformed cristae as well as a 50% decrease in respiratory rate of the cell and an increase in glycolysis [72]. While it is clear that ATP5I expression is important to maintain OXPHOS pathway activity, the specifics of how an increase in ATP5I expression could help compensate for limited oxygen remain unclear and warrant further investigation.

In this study we also observed a significant increase in the percentage of heterophils and a significant decrease in the percentage of lymphocytes in the 2,000 m and 4,000 m samples compared to the sea-level samples (Fig 4). A higher neutrophil-lymphocyte ratio in humans (equivalent to the heterophil-lymphocyte ratio in species that have heterophils rather than neutrophils) is used as an indicator of systemic inflammation. High neutrophils can indicate inflammation and low lymphocytes can indicate poor general health or physiological stress [36]. Given the numerous possible causes for these observed changes in white blood cell composition, it is hard to say if and how exactly the hypoxic conditions could be driving this change, but it is apparent that these changes are triggered in the 2,000 m sampling, suggesting that they are independent of the gene expression changes which are not observed until the 4,000 m sampling.

Although not statistically significant, Hb did generally increase throughout the treatment in both individual C and 54, the only two individuals for which we were able to measure Hb using both the HemoCue and the i-stat device at all three elevations (Table 3). An increase in Hb concentration, considered to be somewhat maladaptive, is observed in low-land humans when experiencing hypoxia as well as high-elevation native Andeans, but is not observed in high-elevation native Tibetans or Ethiopians [34, 73, 74]. Additionally, the high-elevation pikas, *O*. *curzoniae*, have been observed to have Hb concentrations much lower than those observed at any elevation in the current study, with Hb g/dl = 10/7 ± .031 [75]. The higher Hb concentrations observed in *O*. *dauurica* along with the trend of increased Hb concentration with increased hypoxic stress suggests that *O*. *dauurica*, although from the same clade as *O*. *curzoniae*, may have different physiological adaptions to deal with hypoxic stress, just as we see different avenues of adaptation between high-elevation human populations. This could mean that while some responses to hypoxic stress may be conserved across pika species (such as up-regulation of genes in the OXPHOS pathway), other responses may differ between species (such as Hb concentration).

## Conclusions

This is the first study to assess gene expression changes within an individual in response to high-elevation hypoxic stress in any non-human organism. With this novel experimental procedure, we have added evidence to corroborate previous pika transcriptomic work that the OXPHOS pathway as well as GO categories taking part in the electron transport chain are being up-regulated in pikas at higher elevations. This study demonstrates that this up-regulation can not only occur within an individual, but within a five-day period. This is a time frame that is vastly shorter than that needed for genetic adaptions, which takes many generations, and thus is a mechanism that can easily keep pace with rapid Anthropogenic climate change. Thus, our study adds to evidence that acclimation, through changes in gene expression, could be a mechanism that pikas have at their disposal to allow them to occupy vastly different elevations, in this case, 0 to 4,000 m. These findings suggest that individual pikas potentially have an acclimation capacity to tolerate hypoxia levels characteristic of any elevation within their species range. Thus, it does not appear that hypoxia alone should limit dispersal in pikas as they experience range contractions to higher elevations due to climate change. However, while these results suggest a rapid upslope dispersal ability in pikas, the potential long-term fitness effects that hypoxia may have on them have yet to be explored. Additionally, it is important to note that there are numerous other factors that could limit the ability of pikas to move to higher elevations within their species’ distribution even if hypoxia tolerance is not one of them. Some of these factors already described in pikas are fragmented habitat and barriers to dispersal [76, 77] as well as climate-change driven changes in the elevational distribution of the plants they depend on for food [78]. Thus, while we find pikas to be resilient to changes in hypoxia, this does not mean that they will be resilient to the multiple other challenges associated with climate change induced range shifts.

## Supporting information

S1 Fig The daily weight in grams of each of the pikas throughout the experiment. There is no weight data for the first two days of the experiment.
(DOCX)

S2 Fig Percent overlap of leading edge genes between gene sets for 4,000 m vs all other samples comparison. The percent overlap is given in each cell and indicated by shading of the cell.
(DOCX)

S3 Fig Heat map of gene expression for transcripts from the oxidative phosphorylation pathway gene set that are up-regulated in the 4,000 m samples compared to all other samples. Each row is a sample with baseline samples indicated in black, sea-level samples indicated in grey, 2,000 m samples indicated in teal, and 4,000 m samples indicated in dark blue. In the heat map itself, lower expression is indicated in yellows and higher expression is indicated in blues. The mean number of DESeq normalized reads for each transcript is indicated along the top horizontal.
(DOCX)

S4 Fig Heat map of gene expression for transcripts from the oxidative phosphorylation pathway gene set that are up-regulated in the 4,000 m samples compared to all other samples with sample 54 at 4,000 m excluded. Each row is a sample with baseline samples indicated in black, sea-level samples indicated in grey, 2,000 m samples indicated in teal, and 4,000 m samples indicated in dark blue. In the heat map itself, lower expression is indicated in yellows and higher expression is indicated in blues. The mean number of DESeq normalized reads for each transcript is indicated along the top horizontal.
(DOCX)

S5 Fig GSEA normalized enrichment score results for each gene set when excluding sample 54 at 4,000 m. A diagram in the lower right of each panel indicates which sampling groups are being compared. In all comparisons, the higher-elevation group is being compared to the lower-elevation group(s), so positive normalized enrichment scores indicate gene sets that are positively enriched in the higher-elevation samples compared to the lower-elevations samples. A) 4,000 m samples (excluding 54) compared to all other samples. B) 4,000 m samples (excluding 54) compared to baseline and sea-level samples. Gene sets drawn from the pika study are shown in blue, gene sets drawn from the deer mouse studies are shown in grey, and gene sets included due to their GO category definition are shown in purple. The oxidative phosphorylation pathway, which is drawn from both the pika and deer mouse studies is shown in blue and grey stripes. FDR q-values are indicated (*p<0.05).
(DOCX)

S1 Table GSEA results for 2,000 m samples vs. baseline and sea-level samples. (DOCX)

S2 Table GSEA results for 4,000 m samples vs. all other samples. (DOCX)

S3 Table GSEA results for 4,000 m samples vs. baseline and sea-level samples. (DOCX)

S4 Table GSEA results for 4,000 m samples (excluding 54) vs. all other samples. (DOCX)

S5 Table GSEA results for 4,000 m samples (excluding 54) vs. baseline and sea-level samples. (DOCX)
