# Supplementary material for: Experimental study of hypoxia-induced changes in gene expression in an Asian pika, Ochotona dauurica
Source: PLoS One. 2020 Oct 12;15(10):e0240435. doi: 10.1371/journal.pone.0240435 (PMC7549823; doi:10.1371/journal.pone.0240435)
Supplement: S1 Fig — There is no weight data for the first two days of the experiment. (DOCX) [file pone.0240435.s001.docx]

**S1 Fig.** **The daily weight in grams of each of the pikas throughout the experiment.** There is no weight data for the first two days of the experiment.
