# Supplementary material for: Experimental study of hypoxia-induced changes in gene expression in an Asian pika, Ochotona dauurica
Source: PLoS One. 2020 Oct 12;15(10):e0240435. doi: 10.1371/journal.pone.0240435 (PMC7549823; doi:10.1371/journal.pone.0240435)
Supplement: S2 Fig — The percent overlap is given in each cell and indicated by shading of the cell. (DOCX) [file pone.0240435.s002.docx]

**S2 Fig. Percent overlap of leading edge genes between gene sets for 4,000 m vs all other samples comparison.** The percent overlap is given in each cell and indicated by shading of the cell.
