# Supplementary material for: Experimental study of hypoxia-induced changes in gene expression in an Asian pika, Ochotona dauurica
Source: PLoS One. 2020 Oct 12;15(10):e0240435. doi: 10.1371/journal.pone.0240435 (PMC7549823; doi:10.1371/journal.pone.0240435)
Supplement: S3 Fig — Each row is a sample with baseline samples indicated in black, sea-level samples indicated in grey, 2,000 m samples indicated in teal, and 4,000 m samples indicated in dark blue. In the heat map itself, lower expression is indicated in yellows and higher expression is indicated in blues. The mean number of DESeq normalized reads for each transcript is indicated along the top horizontal. (DOCX) [file pone.0240435.s003.docx]

**S3 Fig.** **Heat map of gene expression for transcripts from the oxidative phosphorylation pathway gene set that are up-regulated in the 4,000 m samples compared to all other samples.** Each row is a sample with baseline samples indicated in black, sea-level samples indicated in grey, 2,000 m samples indicated in teal, and 4,000 m samples indicated in dark blue. In the heat map itself, lower expression is indicated in yellows and higher expression is indicated in blues. The mean number of DESeq normalized reads for each transcript is indicated along the top horizontal.
