# Supplementary material for: Experimental study of hypoxia-induced changes in gene expression in an Asian pika, Ochotona dauurica
Source: PLoS One. 2020 Oct 12;15(10):e0240435. doi: 10.1371/journal.pone.0240435 (PMC7549823; doi:10.1371/journal.pone.0240435)
Supplement: S5 Fig — A diagram in the lower right of each panel indicates which sampling groups are being compared. In all comparisons, the higher-elevation group is being compared to the lower-elevation group(s), so positive normalized enrichment scores indicate gene sets that are positively enriched in the higher-elevation samples compared to the lower-elevations samples. A) 4,000 m samples (excluding 54) compared to all other samples. B) 4,000 m samples (excluding 54) compared to baseline and sea-level samples. Gene sets drawn from the pika study are shown in blue, gene sets drawn from the deer mouse studies are shown in grey, and gene sets included due to their GO category definition are shown in purple. The oxidative phosphorylation pathway, which is drawn from both the pika and deer mouse studies is shown in blue and grey stripes. FDR q-values are indicated (*p<0.05). (DOCX) [file pone.0240435.s005.docx]

**S5 Fig. GSEA normalized enrichment score results for each gene set when excluding sample 54 at 4,000 m.** A diagram in the lower right of each panel indicates which sampling groups are being compared. In all comparisons, the higher-elevation group is being compared to the lower-elevation group(s), so positive normalized enrichment scores indicate gene sets that are positively enriched in the higher-elevation samples compared to the lower-elevations samples. A) 4,000 m samples (excluding 54) compared to all other samples. B) 4,000 m samples (excluding 54) compared to baseline and sea-level samples. Gene sets drawn from the pika study are shown in blue, gene sets drawn from the deer mouse studies are shown in grey, and gene sets included due to their GO category definition are shown in purple. The oxidative phosphorylation pathway, which is drawn from both the pika and deer mouse studies is shown in blue and grey stripes. FDR q-values are indicated (*p<0.05).
