# Supplementary material for: Experimental study of hypoxia-induced changes in gene expression in an Asian pika, Ochotona dauurica
Source: PLoS One. 2020 Oct 12;15(10):e0240435. doi: 10.1371/journal.pone.0240435 (PMC7549823; doi:10.1371/journal.pone.0240435)
Supplement: S1 Table — (DOCX) [file pone.0240435.s006.docx]

**S1 Table. GSEA results for 2,000 m samples vs. baseline and sea-level samples.**

| **Gene set** | **# of transcripts** | **ES** | **NES** | **FDR q-val** |
| --- | --- | --- | --- | --- |
| Oxidative phosphorylation (KEGG) | 168 | 0.25 | 1.11 | 1.00 |
| Mitochondrial respiratory chain complex I assembly (GO) | 79 | 0.27 | 1.02 | 1.00 |
| Mitochondrial electron transport, NADH to ubiquinone (GO) | 71 | 0.26 | 0.97 | 1.00 |
| Negative regulation of vascular permeability (GO) | 15 | 0.33 | 0.87 | 1.00 |
| Mitochondrial respiratory chain complex I (GO) | 66 | 0.22 | 0.84 | 1.00 |
| Water transport (GO) | 22 | 0.27 | 0.81 | 0.89 |
| Cellular response to hypoxia (GO) | 215 | -0.20 | -0.75 | 0.96 |
| Angiogenesis (GO) | 588 | -0.20 | -0.81 | 0.96 |
| Regulation of ERK1 and ERK2 cascade (GO) | 342 | -0.21 | -0.84 | 0.98 |
| Muscle structure development (GO) | 980 | -0.22 | -0.90 | 0.89 |
| Response to oxidative stress (GO) | 600 | -0.22 | -0.92 | 0.89 |
| Response to hypoxia (GO) | 448 | -0.24 | -0.96 | 0.86 |
| Mitochondrial inner membrane (GO) | 595 | -0.23 | -0.96 | 0.94 |
| NADH dehydrogenase (ubiquinone) activity (GO) | 57 | -0.30 | -0.97 | 0.99 |
| Cellular response to reactive oxygen species (GO) | 216 | -0.26 | -1.01 | 0.89 |
| Regulation of skeletal muscle cell differentiation (GO) | 30 | -0.35 | -1.01 | 1.00 |
| Regulating of erythrocyte differentiation (GO) | 56 | -0.32 | -1.03 | 1.00 |
| Cellular response to oxidative stress (GO) | 380 | -0.26 | -1.05 | 1.00 |
| Fatty acid oxidation (KEGG) | 15 | -0.45 | -1.10 | 0.96 |
| Lipid catabolic process (GO) | 347 | -0.28 | -1.10 | 1.00 |
| HIF-1 signaling pathway (KEGG) | 176 | -0.31 | -1.19 | 0.91 |
| Notch signaling pathway (GO) | 239 | -0.32 | -1.22 | 1.00 |
