# Supplementary material for: Experimental study of hypoxia-induced changes in gene expression in an Asian pika, Ochotona dauurica
Source: PLoS One. 2020 Oct 12;15(10):e0240435. doi: 10.1371/journal.pone.0240435 (PMC7549823; doi:10.1371/journal.pone.0240435)
Supplement: S2 Table — (DOCX) [file pone.0240435.s007.docx]

**S2 Table. GSEA results for 4,000 m samples vs. all other samples.**

| **Gene set** | **# of transcripts** | **ES** | **NES** | **FDR q-val** |
| --- | --- | --- | --- | --- |
| Oxidative phosphorylation (KEGG) | 168 | 0.46 | 1.99 | 0.001** |
| Mitochondrial inner membrane (GO) | 595 | 0.38 | 1.84 | 0.005** |
| Mitochondrial electron transport, NADH to ubiquinone (GO) | 71 | 0.47 | 1.81 | 0.006** |
| Mitochondrial respiratory chain complex I assembly (GO) | 79 | 0.46 | 1.80 | 0.004** |
| Mitochondrial respiratory chain complex I (GO) | 66 | 0.47 | 1.76 | 0.004** |
| NADH dehydrogenase (ubiquinone) activity (GO) | 57 | 0.46 | 1.64 | 0.008** |
| Cellular response to hypoxia (GO) | 215 | 0.26 | 1.16 | 0.21 |
| Cellular response to reactive oxygen species (GO) | 216 | 0.24 | 1.08 | 0.32 |
| Cellular response to oxidative stress (GO) | 380 | 0.22 | 1.06 | 0.33 |
| Response to oxidative stress (GO) | 600 | 0.21 | 1.04 | 0.33 |
| Regulation of skeletal muscle cell differentiation (GO) | 30 | -0.21 | -0.64 | 0.99 |
| Notch signaling pathway (GO) | 239 | -0.24 | -0.99 | 0.52 |
| Response to hypoxia (GO) | 448 | -0.23 | -1.00 | 0.55 |
| Lipid catabolic process (GO) | 347 | -0.26 | -1.11 | 0.28 |
| Muscle structure development (GO) | 980 | -0.25 | -1.11 | 0.31 |
| Fatty acid oxidation (KEGG) | 15 | -0.46 | -1.13 | 0.31 |
| HIF-1 signaling pathway (KEGG) | 176 | -0.29 | -1.16 | 0.29 |
| Regulation of ERK1 and ERK2 cascade (GO) | 342 | -0.28 | -1.18 | 0.29 |
| Regulating of erythrocyte differentiation (GO) | 56 | -0.36 | -1.21 | 0.28 |
| Angiogenesis (GO) | 588 | -0.31 | -1.38 | 0.09 |
| Negative regulation of vascular permeability (GO) | 15 | -0.58 | -1.44 | 0.08 |
| Water transport (GO) | 22 | -0.57 | -1.55 | 0.055 |
