# Supplementary material for: Experimental study of hypoxia-induced changes in gene expression in an Asian pika, Ochotona dauurica
Source: PLoS One. 2020 Oct 12;15(10):e0240435. doi: 10.1371/journal.pone.0240435 (PMC7549823; doi:10.1371/journal.pone.0240435)
Supplement: S3 Table — (DOCX) [file pone.0240435.s008.docx]

**S3 Table. GSEA results for** **4,000 m samples vs. baseline and sea-level samples.**

| **Gene set** | **# of transcripts** | **ES** | **NES** | **FDR q-val** |
| --- | --- | --- | --- | --- |
| Mitochondrial respiratory chain complex I assembly (GO) | 79 | 0.51 | 1.99 | 0.002** |
| Oxidative phosphorylation (KEGG) | 168 | 0.44 | 1.88 | 0.001** |
| Mitochondrial respiratory chain complex I (GO) | 66 | 0.48 | 1.76 | 0.004** |
| Mitochondrial inner membrane (GO) | 595 | 0.35 | 1.71 | 0.004** |
| Mitochondrial electron transport, NADH to ubiquinone (GO) | 71 | 0.46 | 1.71 | 0.004** |
| NADH dehydrogenase (ubiquinone) activity (GO) | 57 | 0.46 | 1.65 | 0.005** |
| Cellular response to hypoxia (GO) | 215 | 0.26 | 1.16 | 0.19 |
| Cellular response to reactive oxygen species (GO) | 216 | 0.23 | 1.00 | 0.47 |
| Response to oxidative stress (GO) | 600 | 0.20 | 0.98 | 0.50 |
| Regulation of skeletal muscle cell differentiation (GO) | 30 | -0.25 | -0.73 | 0.97 |
| Cellular response to oxidative stress (GO) | 380 | -0.21 | -0.86 | 0.90 |
| Response to hypoxia (GO) | 448 | -0.24 | -0.99 | 0.60 |
| Notch signaling pathway (GO) | 239 | -0.26 | -1.05 | 0.44 |
| Muscle structure development (GO) | 980 | -0.25 | -1.08 | 0.40 |
| Regulation of ERK1 and ERK2 cascade (GO) | 342 | -0.27 | -1.09 | 0.42 |
| HIF-1 signaling pathway (KEGG) | 176 | -0.29 | -1.09 | 0.46 |
| Regulating of erythrocyte differentiation (GO) | 56 | -0.36 | -1.19 | 0.24 |
| Lipid catabolic process (GO) | 347 | -0.29 | -1.19 | 0.27 |
| Water transport (GO) | 22 | -0.46 | -1.23 | 0.24 |
| Fatty acid oxidation (KEGG) | 15 | -0.51 | -1.25 | 0.27 |
| Negative regulation of vascular permeability (GO) | 15 | -0.52 | -1.26 | 0.40 |
| Angiogenesis (GO) | 588 | -0.30 | -1.28 | 0.65 |
