# Supplementary material for: Experimental study of hypoxia-induced changes in gene expression in an Asian pika, Ochotona dauurica
Source: PLoS One. 2020 Oct 12;15(10):e0240435. doi: 10.1371/journal.pone.0240435 (PMC7549823; doi:10.1371/journal.pone.0240435)
Supplement: S4 Table — (DOCX) [file pone.0240435.s009.docx]

**S4 Table. GSEA results for** **4,000 m samples (excluding 54) vs. all other samples.**

| **Gene set** | **# of transcripts** | **ES** | **NES** | **FDR q-val** |
| --- | --- | --- | --- | --- |
| Mitochondrial inner membrane (GO) | 595 | 0.37 | 1.68 | 0.022* |
| Mitochondrial respiratory chain complex I assembly (GO) | 79 | 0.45 | 1.64 | 0.016* |
| Oxidative phosphorylation (KEGG) | 168 | 0.36 | 1.46 | 0.047* |
| NADH dehydrogenase (ubiquinone) activity (GO) | 57 | 0.41 | 1.39 | 0.060 |
| Mitochondrial respiratory chain complex I (GO) | 66 | 0.39 | 1.37 | 0.056 |
| Mitochondrial electron transport, NADH to ubiquinone (GO) | 71 | 0.37 | 1.31 | 0.071 |
| Regulation of skeletal muscle cell differentiation (GO) | 30 | 0.34 | 1.02 | 0.57 |
| Response to oxidative stress (GO) | 600 | 0.21 | 0.98 | 0.63 |
| Cellular response to hypoxia (GO) | 215 | 0.23 | 0.95 | 0.66 |
| Cellular response to oxidative stress (GO) | 380 | 0.20 | 0.88 | 0.80 |
| Cellular response to reactive oxygen species (GO) | 216 | -0.21 | -0.85 | 0.87 |
| Notch signaling pathway (GO) | 239 | -0.22 | -0.90 | 0.82 |
| Regulation of ERK1 and ERK2 cascade (GO) | 342 | -0.21 | -0.91 | 0.89 |
| Fatty acid oxidation (KEGG) | 15 | -0.38 | -0.93 | 0.90 |
| Regulating of erythrocyte differentiation (GO) | 56 | -0.29 | -0.99 | 0.72 |
| Lipid catabolic process (GO) | 347 | -0.23 | -0.99 | 0.82 |
| Response to hypoxia (GO) | 448 | -0.25 | -1.08 | 0.49 |
| Muscle structure development (GO) | 980 | -0.25 | -1.15 | 0.33 |
| Angiogenesis (GO) | 588 | -0.28 | -1.27 | 0.17 |
| HIF-1 signaling pathway (KEGG) | 176 | -0.32 | -1.30 | 0.17 |
| Negative regulation of vascular permeability (GO) | 15 | -0.54 | -1.36 | 0.17 |
| Water transport (GO) | 22 | -0.52 | -1.40 | 0.25 |
